# The Impact of Maternal COVID-19 Severity on Obstetrical and Neonatal Outcomes: A Retrospective Study

**DOI:** 10.7759/cureus.97597

**Published:** 2025-11-23

**Authors:** Hadi Fakih, Mohammad Ali Ahmad, Hussein Tarhini

**Affiliations:** 1 Pediatrics, Faculty of Medical Sciences, Lebanese University, Beirut, LBN; 2 Pediatrics, Sheikh Ragheb Harb University Hospital, Nabatieh, LBN; 3 Pediatrics, Faculty of Medicine, Lebanese University, Beirut, LBN; 4 Radiology, Faculty of Medicine, Lebanese University, Beirut, LBN

**Keywords:** cesarean section, covid-19, neonatal outcomes, nicu, pregnancy, preterm birth, sars-cov-2

## Abstract

Introduction: Pregnancy induces physiological and immunological changes that increase susceptibility to severe outcomes from viral respiratory infections. Global meta-analyses have established that coronavirus disease 2019 (COVID-19) in pregnancy is associated with significantly elevated risks of adverse outcomes, including maternal ICU admission and preterm birth. However, region-specific data from Lebanon and the Middle East are scarce. This study aimed to evaluate the association between COVID-19 severity and obstetrical/neonatal outcomes in a Lebanese cohort.

Materials and methods: A retrospective, observational, monocentric study was conducted at Sheikh Ragheb Harb University Hospital. We included 110 hospitalized pregnant women with PCR-confirmed COVID-19 and their neonates admitted during 2021. The primary outcome was a composite of adverse neonatal outcomes (neonatal COVID-19 positivity or NICU admission). Secondary outcomes included individual neonatal components (birth weight, APGAR scores) and maternal outcomes (mode of delivery, preterm birth): statistical analyses employed chi-square tests, t-tests, and univariate logistic regression.

Results: Most patients (86.4%, n=95) had asymptomatic/mild COVID-19, while 13.6% (n=15) had moderate/severe disease. Moderate/severe maternal COVID-19 was significantly associated with an increased likelihood of cesarean delivery (odds ratio (OR)=4.70, 95% confidence interval (CI) 1.51-14.60, p=0.011) and preterm birth (OR=3.74, 95% CI 1.20-11.67, p=0.040). Neonates born to mothers with severe COVID-19 had significantly higher odds of testing PCR-positive (OR=27.00, 95% CI 6.86-106.30, p<0.001), requiring NICU admission (OR=14.33, 95% CI 4.15-49.55, p<0.001), and having lower APGAR scores (OR=0.06, 95% CI 0.02-0.25, p<0.001). Severe disease was also associated with lower neonatal birth weight (p=0.009) and prolonged maternal and neonatal hospitalization (p<0.001 for both).

Conclusions: In this Lebanese cohort, maternal COVID-19 severity demonstrated strong associations with adverse obstetrical and neonatal outcomes. The markedly elevated risks, particularly for neonatal infection and NICU admission, underscore the need for vigilant management of pregnant women with COVID-19. However, the observational design, single-center setting, and small number of severe cases necessitate cautious interpretation. These findings highlight the critical importance of preventative strategies, including vaccination, and call for larger, prospective regional studies.

## Introduction

The coronavirus disease 2019 (COVID-19) pandemic, caused by the severe acute respiratory syndrome coronavirus 2 (SARS-CoV-2), has posed unprecedented challenges to global healthcare systems [1]. Pregnant women represent a uniquely vulnerable population due to well-documented physiological and immunological adaptations of pregnancy, including altered cell-mediated immunity, elevated diaphragm, and increased oxygen consumption, which are known to increase susceptibility to severe respiratory infections [2,3].

A substantial body of evidence has quantified the risks associated with COVID-19 in pregnancy. Large-scale meta-analyses and multinational cohorts have consistently reported that infected pregnant women face a significantly increased risk of adverse outcomes compared to their non-infected peers, including a heightened likelihood of intensive care unit (ICU) admission, pre-eclampsia, and maternal mortality [4,5]. From a neonatal perspective, strong associations have been established with preterm birth, low birth weight, and increased rates of neonatal intensive care unit (NICU) admission [5,6]. The mode of delivery has also been extensively investigated, with meta-analyses indicating a higher prevalence of cesarean sections, often attributed to both obstetric complications and, particularly early in the pandemic, precautionary institutional policies [7,8].

While the global literature is robust, data from specific regions, including Lebanon and the broader Middle East, are limited. The Lebanese healthcare system, already strained by concurrent economic and political crises, faced additional pressure during the pandemic, which may have influenced clinical pathways and resource allocation. Understanding the local epidemiology and clinical impact of SARS-CoV-2 infection in pregnant women is crucial for developing effective public health and clinical management strategies tailored to this setting. This study aimed to bridge this knowledge gap by evaluating the clinical outcomes of hospitalized pregnant patients with COVID-19 and their offspring at a university hospital in South Lebanon. We hypothesized that the severity of maternal COVID-19 infection would be significantly associated with worse obstetrical and neonatal outcomes.

## Materials and methods

Study design and population

This was a retrospective, observational, monocentric cohort study conducted at Sheikh Ragheb Harb University Hospital in South Lebanon. The study population consisted of all hospitalized pregnant women with a confirmed COVID-19 diagnosis via polymerase chain reaction (PCR) testing and their neonates, who were admitted during the 2021 calendar year. This design intentionally captured a cohort with more severe disease requiring hospitalization.

Ethical considerations

The study protocol was reviewed and approved by the Institutional Review Board of Sheikh Ragheb Harb University Hospital (approval code: IRB23RP14; date: May 29, 2023). Given the study's retrospective nature, informed consent was waived. All patient data were anonymized and de-identified prior to analysis to ensure confidentiality and compliance with Good Clinical Practice guidelines.

Inclusion and exclusion criteria

Inclusion criteria were (1) hospitalized pregnant women aged 18 years or older; (2) gestational age of 24 weeks or more; (3) a positive COVID-19 PCR test at the time of hospitalization; and (4) delivery during the same hospitalization. Exclusion criteria included (1) maternal age under 18 years; (2) gestational age less than 24 weeks; (3) pre-existing maternal comorbidities such as chronic hypertension or pre-gestational diabetes (to minimize confounding); and (4) neonates with multiple congenital malformations.

Data collection

Data were extracted retrospectively from hospital medical records using a standardized data collection form. Collected maternal variables included age, parity (primigravida/multiparous), weight, height, body mass index (BMI), COVID-19 severity, mode of delivery (vaginal/cesarean), gestational age at delivery, and length of hospital stay (LOS). COVID-19 severity was classified, for analytical purposes, into two categories according to the National Institutes of Health criteria: "asymptomatic/mild" and "moderate/severe" [9].

Neonatal variables included birth weight, gestational age at birth (with preterm defined as <37 weeks), neonatal COVID-19 status (determined by PCR testing from a nasopharyngeal swab taken within the first 24 hours of life), NICU admission, neonatal LOS, and APGAR scores at one and five minutes. In line with hospital policy at the time, neonates were separated from SARS-CoV-2-positive mothers immediately after birth. NICU admission was based on the presence of clinical manifestations (e.g., respiratory distress, prematurity requiring support) and not solely for precautionary observation.

Statistical analysis

Data were analyzed using the SPSS Statistics version 26.0 (IBM Corp. Released 2019. IBM SPSS Statistics for Windows, Version 26.0. Armonk, NY: IBM Corp.). Descriptive statistics were computed for all variables. Categorical variables were presented as frequencies and percentages, while continuous variables were summarized as means with standard deviations (SD) or medians with interquartile ranges (IQR). The association between COVID-19 severity and categorical outcomes was assessed using the chi-square test or Fisher’s exact test, as appropriate. For continuous variables, the independent-samples t-test or the Mann-Whitney U test was used. Binary logistic regression was used to estimate unadjusted odds ratios (ORs) with 95% confidence intervals (CIs) to quantify the association between COVID-19 severity and key dichotomous outcomes. A p-value of less than 0.05 was considered statistically significant for all tests. For univariate logistic regression models, goodness-of-fit was assessed using the Hosmer-Lemeshow test. Given the sample size and the focus on a single predictor, multivariable adjustment was not performed.

## Results

Demographic and clinical characteristics

A total of 110 pregnant women with COVID-19 and their corresponding neonates were included in the analysis. The demographic and clinical characteristics of the study population are summarized in Table 1. The mean age of the participants was 26.97 years (SD=5.02), and the majority were multiparous (79.1%, n=87). The mean BMI was 29.45 kg/m² (SD=4.94), indicating a predominantly overweight cohort. Regarding delivery outcomes, most women had a vaginal delivery (70.9%, n=78), with a mean gestational age at delivery of 38.17 weeks (SD=1.33). The average maternal LOS was 1.85 days (SD=0.89).

**Table 1:** Baseline demographic and clinical characteristics of the study population (N=110) SD: standard deviation, BMI: body mass index, COVID-19: coronavirus disease 2019, LOS: length of hospital stay

Characteristic	Value
Age (years), mean (SD)	26.97 (5.02)
Parity, n (%)	
Primigravida	23 (20.9%)
Multiparous	87 (79.1%)
BMI (kg/m²), mean (SD)	29.45 (4.94)
COVID-19 severity, n (%)	
Asymptomatic/mild	95 (86.4%)
Moderate/severe	15 (13.6%)
Mode of delivery, n (%)	
Vaginal	78 (70.9%)
Cesarean section	32 (29.1%)
Gestational age (weeks), mean (SD)	38.17 (1.33)
Maternal LOS (days), mean (SD)	1.85 (0.89)

Analysis of neonatal outcomes (Table 2) revealed a preterm birth rate of 22.7% (n=25). The mean neonatal birth weight was 3204.86 grams (SD=431.05). Neonatal COVID-19 positivity was observed in 12.7% (n=14) of infants, and 16.4% (n=18) required NICU admission. The vast majority of neonates (89.1%, n=98) had APGAR scores of 9 or 10 at one and five minutes.

**Table 2:** Neonatal outcomes (N=110) SD: standard deviation, COVID-19: coronavirus disease 2019, NICU: neonatal intensive care unit, LOS: length of hospital stay

Characteristic	Value
Preterm birth (<37 weeks), n (%)	25 (22.7%)
Birth weight (grams), mean (SD)	3204.86 (431.05)
Neonatal COVID-19 positive, n (%)	14 (12.7%)
NICU admission, n (%)	18 (16.4%)
Neonatal LOS (days), mean (SD)	2.05 (1.17)
APGAR score (1-5 min), n (%)	
8-9	12 (10.9%)
9-10	98 (89.1%)

Association between COVID-19 severity and outcomes

No significant associations were found between COVID-19 severity and baseline demographic characteristics, including parity, age, or BMI (all p>0.05).

Maternal Outcomes

A significant association was observed between disease severity and the mode of delivery (p=0.011). Logistic regression analysis (Table 3) indicated that mothers with moderate/severe COVID-19 had 4.70 times higher odds of undergoing a cesarean section compared to those with asymptomatic/mild disease (95% CI (1.51-14.60)). Furthermore, maternal LOS was significantly longer in the moderate/severe group (mean: 3.40 days, SD=0.74) compared to the asymptomatic/mild group (mean: 1.60 days, SD=0.63; p<0.001).

**Table 3:** Effect of COVID-19 severity on key maternal and neonatal outcomes (binary logistic regression) COVID-19: coronavirus disease 2019, NICU: neonatal intensive care unit, OR: odds ratio, CI: confidence interval

Outcome	Asymptomatic/mild (n=95)	Moderate/severe (n=15)	OR	95% CI for OR	p-value
Cesarean delivery, n (%)	23 (24.2%)	9 (60.0%)	4.696	1.510-14.604	0.011
Preterm birth, n (%)	18 (18.9%)	7 (46.7%)	3.743	1.201-11.666	0.04
Neonatal COVID-19+, n (%)	5 (5.3%)	9 (60.0%)	27	6.858-106.299	<0.001
NICU admission, n (%)	9 (9.5%)	9 (60.0%)	14.333	4.146-49.550	<0.001
APGAR score 9-10, n (%)	90 (94.7%)	8 (53.3%)	0.063	0.016-0.246	<0.001

Neonatal Outcomes

The impact of maternal disease severity on neonatal outcomes was profound. Neonates born to mothers with moderate/severe COVID-19 had significantly lower mean birth weights (2937.33 g vs. 3247.11 g, p=0.009) and longer LOS (mean: 3.73 days vs. 1.79 days, p<0.001). As detailed in Table 3, these neonates were at substantially higher risk for adverse outcomes: they were 3.74 times more likely to be born preterm (95% CI (1.20-11.67)), 27.00 times more likely to test positive for COVID-19 (95% CI (6.86-106.30)), and 14.33 times more likely to be admitted to the NICU (95% CI (4.15-49.55)). Additionally, they were significantly less likely to achieve a high APGAR score (9-10) (OR=0.06, 95% CI (0.02-0.25), p<0.001).

## Discussion

This retrospective cohort study provides critical insights into the association between maternal COVID-19 severity and obstetrical and neonatal outcomes in a Lebanese population. Our findings demonstrate strong and significant associations between moderate to severe COVID-19 and an increased risk of cesarean delivery, preterm birth, neonatal SARS-CoV-2 positivity, NICU admission, lower birth weight, and suboptimal APGAR scores.

The strong association between severe COVID-19 and cesarean section (OR=4.70) is consistent with the global literature [7, 8]. In our cohort, this was primarily driven by acute maternal (e.g., respiratory distress) and fetal (e.g., non-reassuring status) medical indications directly related to the pathophysiology of severe COVID-19, rather than a blanket institutional policy. The increased rate of preterm birth (OR=3.74) aligns with studies reporting a dose-response relationship between the severity of maternal infection and the risk of early delivery [6, 10]. This is likely multifactorial, involving both iatrogenic delivery to manage a deteriorating maternal condition and spontaneous preterm labor triggered by the systemic inflammatory response associated with severe COVID-19 [5].

A particularly striking finding was the dramatically increased odds of neonatal COVID-19 positivity (OR=27.00) among infants born to mothers with severe disease. Given the hospital policy of immediate mother-neonate separation and PCR testing within 24 hours of birth, this high rate, while not confirming vertical transmission, strongly suggests its possibility and warrants further investigation. The concomitant increase in NICU admissions (OR=14.33), which were based on clinical need and not precaution, underscores the tangible clinical vulnerability and morbidity of these neonates, likely driven by prematurity, the consequences of maternal illness, and potentially the infection itself [11].

The significantly lower APGAR scores and birth weights in the severe maternal COVID-19 group further highlight the detrimental fetal impact of severe maternal illness. Maternal hypoxemia and systemic inflammation can impair placental function and fetal oxygenation, leading to a compromised neonatal condition at birth [12]. The observed difference in birth weight is likely confounded by the higher rate of preterm birth in the severe group. The prolonged hospitalization for both mothers and neonates in the severe group reflects the increased healthcare resource utilization required in these complex cases.

Our study has several significant limitations. Its retrospective and single-center design limits generalizability. The small number of patients with moderate/severe disease (n=15), while reflective of the population distribution, reduces the stability of our statistical models, likely inflating the magnitude of the point estimates (e.g., OR=27.00) and resulting in wide confidence intervals. Excluding hospitalized patients means our findings are not representative of all COVID-19 infections in pregnancy. Furthermore, the absence of data on key potential modifiers, such as maternal vaccination status, SARS-CoV-2 viral variant, and socioeconomic factors, limits a more comprehensive interpretation. Finally, the exclusion of women with pre-existing comorbidities resulted in a healthier cohort with a lower baseline risk, which may limit the applicability of our findings to pregnant populations with a higher prevalence of underlying conditions.

## Conclusions

This study demonstrates strong associations between the severity of maternal COVID-19 and a heightened risk of adverse obstetrical and neonatal outcomes in a Lebanese cohort. The markedly elevated risks for cesarean delivery, preterm birth, neonatal SARS-CoV-2 positivity, and NICU admission underscore the substantial burden of severe COVID-19 in pregnancy and highlight the imperative for vigilant clinical management.

However, the observational design, single-center setting, small sample size in the severe-disease subgroup, and absence of data on key confounders necessitate caution in interpreting these findings as demonstrating association rather than causation. Despite these limitations, this study provides crucial initial evidence from a previously understudied region. It strengthens the argument for robust preventive strategies, including vaccination. It underscores the need for larger, prospective, multicenter studies in the region to confirm these associations, investigate long-term neonatal outcomes, and guide the development of optimized care protocols.
